# Subclinical atherosclerosis and associated risk factors among HIV-infected adults in Jos, North Central Nigeria: a cross-sectional study

**DOI:** 10.11604/pamj.2020.37.388.21073

**Published:** 2020-12-31

**Authors:** Lucius Chidiebere Imoh, Charles Chibunna Ani, Kuleve Othniel Iyua, Alfred Ibu Odo, Ganiyu Adeniyi Amusa, Godwin Osawaru Osaigbovo, Christian Ogoegbulam Isichei, Oche Ochai Agbaji, Godwin Imade, Ayuba Ibrahim Zoakah, Basil Nwaneri Okeahialam, Atiene Solomon Sagay

**Affiliations:** 1 Department of Chemical Pathology, Jos University Teaching Hospital, Plateau State, Nigeria,; 2 Department of Radiology, Jos University Teaching Hospital, Plateau State, Nigeria,; 3 Department of Medicine, Jos University Teaching Hospital, Plateau State, Nigeria,; 4 Department of Obstetrics and Gynaecology, Jos University Teaching Hospital, Plateau State, Nigeria,; 5 Department of Community Medicine, Jos University Teaching Hospital, Plateau State, Nigeria

**Keywords:** HIV, subclinical atherosclerosis, carotid-intima-media-thickness, cardiovascular disease, cardiovascular disease risk factor, metabolic syndrome

## Abstract

**Introduction:**

human immunodeficiency virus (HIV) is evolving into a leading cause of cardiovascular diseases (CVD) in sub-Saharan Africa (SSA) where the burden of HIV remains high. Atherosclerosis underlie progression to CVD. We therefore examined the prevalence of subclinical atherosclerosis and its association with traditional and non-traditional risk factors for CVD in Nigerian HIV-infected adults.

**Methods:**

this was a cross-sectional study involving randomly selected stable HIV-infected patients with undetectable viral load attending HIV clinics at the Jos University Teaching Hospital and Faith Alive Foundation in Jos, Nigeria. Demographic data, biophysical measurements, cardiovascular risk factors and information regarding HIV-related factors, fasting serum lipid profile, fasting plasma glucose, high-sensitivity C-reactive protein and Carotid-Intima-Media-Thickness (CIMT) were assessed. Subclinical atherosclerosis was defined using a cut-off value of mean CIMT ≥ 0.78 mm. Data were analyzed with the Statistical Package for Social Sciences® (SPSS) software version 23.0 (IBM Corp., Chicago, Illinois, USA). Bivariate analysis and multivariate logistic regression were used to examine the association between risk factors of CVD and subclinical atherosclerosis. The statistical significance level was set at p ≤ 0.05.

**Results:**

a total of 148 HIV adults (70.9% being females) on Anti-Retroviral Therapy (ART) were included in this study. The prevalence of subclinical atherosclerosis was 7.4%. Among subjects with subclinical atherosclerosis (SCA), 63.6% were males and 81.8% were hypertensive. Elevated blood glucose, lipids and high-sensitivity C-reactive protein, body mass index (BMI), HIV-related parameters (duration of HIV infection, antiretroviral regimen, CD4+ cell count), current smoking status, alcohol use, were not significantly associated with subclinical atherosclerosis (p>0.05). Male gender [OR(95%CI=4.91(1.36-17.77)], age [OR(95%CI)=1.14(1.06-1.23)], hypertension [OR(95%CI=14.4(3.03-71.86)] and metabolic syndrome [OR(95%CI=8.34(1.73-40.18)] were significantly associated with SCA at bivariate analysis. After adjusting for age, sex and antiretroviral regimen, only increasing age [Adjusted Odds Ratio (AOR) (95% confidence interval (CI)] = 1.12(1.01-1.25)] and hypertension [AOR (95%CI)=10.67 (1.31-87.18)], remained as independent predictors of subclinical atherosclerosis (SCA).

**Conclusion:**

the prevalence of subclinical atherosclerosis among HIV-infected adults is high in Nigeria. It is significantly associated with increasing age and hypertension. Traditional CVD risk factors such as dyslipidaemia, diabetes mellitus and obesity were not associated with subclinical atherosclerosis in this population.

## Introduction

Cardiovascular disease (CVD) is the leading cause of death and morbidity globally [1,2]. HIV is fast gaining reputation as a leading cause of CVDs in sub-Saharan Africa (SSA) [3,4]. In addition to the usual risk factors for CVDs in the general populations, People Living with HIV (PLHIV) may have addition risk factors related to endothelial dysfunction, metabolic effects of antiretroviral drugs such as dyslipidemias, insulin resistance and the chronic inflammation induced by Human Immunodeficiency Virus (HIV) which heighten their predisposition to CVDs [5,6]. The burden of HIV remains high in SSA and Nigeria is among the countries with the highest prevalence with about 3.5 million people infected [7]. Increased burden of non-communicable diseases (NCDs) such as CVDs become more significant in view of increasing life expectancy of PLHIV due to ART [5].

Atherosclerosis is a fundamental vascular disease underlying the development or progression to CVDs [8]. It involves a complex pathophysiologic process resulting in deposition of fatty materials, thickening of the intima and narrowing of blood vessels. The combination of endothelial dysfunction, chronic inflammation and dyslipidemias are central to the pathogenesis of atherosclerosis and these conditions have been documented in HIV-infected patients [5,6,9]. The presence of subclinical atherosclerosis (SCA) which can be assessed by carotid intimal media thickness (CIMT) is a harbinger for CVDs [10-12]. CIMT is a well validated surrogate imaging marker for cardiovascular assessment and future cardiovascular morbidity and mortality risk [12-18]. However, the significance of arterial stiffness and SCA have not been well characterized in Nigerian HIV-infected patients. Few studies in Nigeria have investigated the presence of risk factors of CVDs in PLHIV without relating them to cardiovascular endpoints therefore, it is not clear if these risk factors are actually associated with vascular diseases or a high risk of progression to CVDs [19-21]. There is paucity of data on the prevalence of subclinical atherosclerosis in PLHIV in Nigeria. The aim of this study was to determine the prevalence of subclinical atherosclerosis and the associated risk factors in PLHIV in Jos, North-Central, Nigeria.

## Methods

**Study area**: this cross-sectional study was conducted at the AIDS Prevention Initiative in Nigeria supported HIV clinics at the Jos University Teaching Hospital (JUTH) and Faith Alive Foundation (FAF). The JUTH HIV clinic is located in Jos, Nigeria. It serves a population of over 22 million people from Plateau and the neighboring North Central States of Nigeria. The clinic commenced HIV services in 2004, and has cumulatively enrolled over 26,000 adults for HIV care and treatment; with about 10,000 on follow-up [22]. Faith Alive Foundation is a non-governmental faith-based organization in Jos, Nigeria that provides primary healthcare for about 10,000 patients in a month and ongoing comprehensive care for over 6000 HIV-infected patients, as well as care for emergencies, and opportunistic infections such as tuberculosis and malaria.

**Study population and sampling method**: the study population included HIV-infected adults on ART between ages 20 and 70 years (inclusive) enrolled at JUTH and FAF HIV clinics. A representative sample of 150 consenting HIV-infected adults who met the inclusion criteria were randomly selected using computer- generated numbers from a sampling frame obtained from the register of patients attending the HIV clinics at JUTH and FAF. Selected HIV-infected adults had undetectable viral load (plasma HIV-1 RNA < 20 copies per milliliter) within 1 year of the study. Patients with confirmed CVD (stroke, myocardial infarction, and/or peripheral vascular disease), malignancy and active infection (documented in the medical records), receiving glucocorticoids, growth hormone or other anabolic agents within the past 6 months were excluded from the study. Also, excluded were patients that were very sick, pregnant women and those on current ARV regimen for less than 6 months.

**Study procedures**: the study participants were scheduled for fasting blood sample collection. Participants who could not come on the scheduled date were rescheduled if they gave prior notice to be excused or replaced if no notice was given. Relevant clinical data were obtained from each participant with the aid of a pretested semi-structured questionnaire. The questionnaire was filled by the researcher or trained assistants. The questionnaire addressed the following areas: socio-demographic data, CVD risks factors, previous diseases, co-morbidities and co-medications. Information regarding HIV-related factors were obtained from patients´ records; known duration of HIV infection, start date of ART, current ARV regimen, and previous changes in regimen, hepatitis B or C co-infection and the latest CD4+ cell count and HIV viral load were recorded if done within the last 12 months. Relevant history of CVD risk factors was also sought for, including; lifestyle characteristics (exercise, diet, history of smoking and alcohol use), family history of CVD (hypertension, stroke, myocardial infarction and sudden death) as well as any concurrent medication that might affect CVD risk (anti-hypertensive drugs, non-steroidal anti-inflammatory drugs, and lipid lowering drugs). Biophysical measurements were obtained according to standard procedures. These included measurements of height, weight, waist circumference, hip circumference and blood pressure (BP). Body mass index (BMI) was also calculated.

**Blood sample collection and biochemical analysis**: blood samples were collected in the morning after a 10-12 hour overnight fast. Four millilitres (mls) of blood was drawn into a plain vacutainer for lipid profile; 3mls into a fluoride oxalate vacutainer for glucose assay. The specimens were centrifuged at 4000 revolutions per minute for 10 minutes and the supernatant, plasma or serum separated out. Plasma glucose was measured on the day of sample collection while samples for aliquots for lipid profile assays were stored at -20°C in a well-monitored freezer for one month until the analyses were performed. Fasting plasma glucose concentration was measured using glucose oxidase method. Total cholesterol (TC), triglyceride (TG) and high-density lipoprotein cholesterol (HDLc) were analyzed with standard enzymatic methods, while low density lipoprotein cholesterol (LDLc) was determined by a direct, homogenous assay. The LDLc was measured by direct homogenous assay method to avoid the limitation of the routinely used Friedewald equation [23] which cannot be applied when TG > 400mg/dl as a very likely scenario in HIV patients on ARVs. All assays were analysed on a Roche Cobas C111 analyzer (Roche diagnostics, Germany). Quality control was assured by simultaneous analysis of control specimens.

**Carotid Intima Media Thickness (CIMT) Measurement**: this was done by a trained radiologist using high resolution real time GE Logiq® C5 color doppler ultrasound scanner (2017 model) (GE Healthcare Chicago, Illinois, USA) fitted with 7.5MHz linear probe. The right and left carotid arteries were studied with the subject in supine position, neck was hyperextended with a soft pillow under the shoulders and head was turned to the contralateral side for each scan. The common carotid artery (CCA) was located at its origin and the probe traced along its long axis with multiple scanning angles (anterior and lateral) to the carotid bifurcation (bulb) and further to the proximal 10 mm of the internal carotid artery (ICA). The Intimal Media Thickness (IMT) was taken in the longitudinal plane at the point of maximal thickness on the far wall of the CCA, (within 10 mm proximal to the bifurcation), the bulb and at the ICA (within 10 mm distal to the bifurcation). The machine was frozen and using its caliper markers, the IMT was measured as the distance between the inner echogenic line representing the intima-blood interface and the outer echogenic line representing the media-adventitia junction. Magnification of the image was used to improve accuracy of the caliper placement. Randomly selected participants were rescanned for quality control. The mean of the maximum IMT from the far wall of the distal common carotid artery, the carotid bifurcation and the proximal internal carotid on the right and left sides (mean of 6 sites) was recorded as the mean CIMT according to the protocol used in the Atherosclerosis Risk In Communities (ARIC) study [24,25].

**Working definitions**: obesity was defined as a BMI ≥30; while overweight was defined as BMI between 25-29.9 kg/m^2^ respectively. Hypertension was defined as systolic blood pressure (SBP) ≥140 mmHg and/or diastolic blood pressure (DBP) ≥90 mmHg and/or the use of antihypertensive medication [26]. Impaired fasting glucose was regarded as fasting blood glucose (FBG) between 6.0 to 6.9 mmol/L and diabetes mellitus was determined by medical history of confirmed diabetes mellitus (from patients´ records), fasting plasma glucose (FPG) ≥7.0 mmol/L and/or random plasma glucose ≥11.1 mmol/L. Metabolic syndrome (MetS) was defined according to the International Diabetes Federation (IDF 2005) guidelines as the constellation of at least three abnormalities-abdominal obesity (abdominal circumference >94 cm in males and >80 cm in females), raised blood pressure (SBP ≥130 mmHg and or DBP ≥85 mmHg), FBG ≥5.6 mmol/L or 6.0 to 6.9 mmol/L, triglycerides ≥1.70 mmol/L and HDLc <1.03 mmol/L in males and <1.30 mmol/L in females [26,27]. Sedentary lifestyle was defined as a lack of regular physical exercise (at least 30 minutes three times weekly). Subclinical atherosclerosis was defined using a cut-off value of mean CIMT ≥0.78 mm, based on the previous study which showed that on average, a healthy adult reaches a CIMT of 0.78 mm at the age of 76 years [28].

**Data processing and statistical analysis**: data was entered into Microsoft Excel^®^version 2.0 (Microsoft Corp., Redmond, Washington, USA) and exported to SPSS^®^software version 23.0 (IBM Corp., Chicago, Illinois, USA) for analysis. Descriptive statistics were presented as mean values + standard deviation (SD) or medians with interquartile ranges (IQRs) for non-parametric continuous variables, and proportions (as percentages) for categorical variables. Unpaired students t-test or Mann-Whitney U test were used to test the difference in means or medians of continuous variables between groups. Subclinical atherosclerosis, defined as CIMT ≥0.78 mm, was the primary outcome variable. Predictors of subclinical atherosclerosis were determined using bivariate and multivariate logistic regression. Independent predictors of subclinical cardiovascular disease such as: biophysical measurements, traditional CVD risk factors and the non-traditional CVD risk factors in HIV-infected patients such as duration of HIV, median CD4+ cell count and ARV status were analyzed. Factors with a p-value of < 0.2 at bivariate analysis were included in a stepwise backward model and expressed as odd ratios. The significance level was set at p ≤0.05.

**Ethics approval and consent to participate**: this study was carried out after due approval from the Ethical Committee of Jos University Teaching Hospital and Faith Alive Foundation. Each participant gave written informed consent to take part in the study. Consent for publication for research purpose was taken from the participant.

## Results

A total of 148 HIV adults on ART were included in this study; of which 70.9% where females. The mean (SD) age of the participants was 42.1 (9.4) years and most (60.1%) were 40 years or older. Table 1 summarises the sociodemographic, anthropometric and biochemical characteristics of the participants. The data are compared between male and females. Males with mean (SD) age of 47.4 (8.9) years were significantly older than females with mean (SD) age of 39.8 (8.8) years, p = 0.045. Males were significantly more likely to be hypertensive and current smokers than females, p < 0.05. Concerning HIV-related characteristics, the median (IQR) known duration of HIV infection and duration of ARV use respectively was 5 (3-5) years and 5 (3-9) years respectively. Males were significantly more likely to have longer period of infection but lower period of ARV use. The overall prevalence of subclinical atherosclerosis as defined in this study was 7.4% (16.3% among males; 3.8% among females).

**Table 1:** general characteristics of the study participants by gender

Variables	All participants n=148	Female n=105 (70.9%)	Male n=43 (29.1%)	P-Value
**Demographic data**				
Age (years; mean (SD))	42.1 (9.4)	39.9 (8.8)	47.4 (8.9)	**<0.001**
**Age group (%)**				**0.002**
20-29	13 (8.8)	12 (11.4)	1 (2.3)	
30-39	46 (31.1)	39 (37.1)	7 (16.3)	
40-49	56 (37.8)	38 (36.2)	18 (41.9)	
50-59	26 (17.6)	14 (13.3)	12 (27.9)	
60-69	7 (4.7)	2 (1.9)	5 (11.6)	
**Medical history**				
Hypertension (%)	41 (27.7)	24 (22.9)	17 (39.5)	**0.045**
Diabetes mellitus (%)	5 (3.4)	4 (3.8)	1 (2.3)	1.000
Family history of stroke (%)	11 (7.4)	6 (5.7)	5 (11.6)	0.298
Smoking status (%)	7 (4.7)	1 (1.0)	6 (14.0)	**0.003**
Alcohol (%)	19 (12.8)	11 (10.5)	8 (18.6)	0.187
Sedentary life>	26 (17.6)	16 (15.2)	10 (23.3)	0.245
**Biophysical parameters**				
Systolic BP (SBP) mmHg (mean; SD)	124.4 (20.7)	121.8 (18.3)	130.7 (24.6)	**0.017**
Diastolic BP (SBP) mmHg (mean; SD)	78.9 (10.9)	78.4 (10.7)	80.4 (11.4)	0.303
BMI (kg/m^2^) (median; IQR)	25.4 (22.2-28.5)	25.8 (22.3-29.3)	23.6 (21.4-27.2)	0.059
Fasting plasma glucose (mmol/L) (mean;SD)	5.3 (1.3)	5.2 1.3)	5.3 (1.3)	0.610
Total cholesterol (mmol/L) (mean;SD)	5.0 (1.3)	5.2 1.3)	4.7 (1.3)	0.053
Triglyceride (mmol/L) (mean;SD)	1.2 (0.9)	1.2 (0.9)	1.3 (0.9)	0.602
HDL cholesterol (mmol/L) (mean;SD)	1.0 (0.4)	1.1 (0.3)	1.0 (0.4)	0.142
LDL cholesterol (mmol/L) (mean;SD)	2.5 (0.9)	2.6 (0.9)	2.2 (1.0)	0.059
HsCRP (mg/L) (median; IQR)	2.8 (0.8-5.7)	2.9 (1.0-5.7)	2.6 (0.6-8.3)	0.818
Metabolic syndrome (IDF) (%)	57 (38.5)	39 (37.1)	18 (41.9)	0.592
Mean CIMT (mean;SD)	0.59 (0.11)	0.57 (0.10)	0.64 (0.12)	**0.001**
Mean IMT (CCA and BULB) (mean;SD)	0.67 (0.14)	0.65 (0.13)	0.73 (0.15)	**0.001**
SCA by Mean CIMT (%)	11 (7.4)	4 (3.8)	7 (16.3)	**0.014**
SCA by Mean IMT (CCA and BULB) (%)	27 (18.2)	15 (14.3)	12 (27.9)	0.062
**HIV-Related parameters**				
Known duration of infection (years) (median; IQR)	5.0 (3.0 -5.0)	5.0 (3.0 -9.0)	8.0 (4.0 -11.0)	**0.024**
Duration of ARV (years) (median; IQR)	5.0 (3.0 -9.0)	7.0 (4.0-11.0))	3.0 (3.0 -5.0)	**<0.001**
Receiving first-line ARV (%)	138 (93.2)	98 (93.3)	40 (93.0)	1.000
Receiving second-line ARV (%)	10 (6.8)	7 (6.7)	3 (7.0)	
Nadir CD4 count (cells/ul) (median; IQR)	168.0 (100.5-244.5)	198.0 (94.5-258.5)	146.0 (102.0-223.0)	0.374
Latest CD4 count (cells/ul) (median; IQR)	448.0 (307.3-607.5)	495.0 (334.0-677.5)	375.0 (287.0-489.0)	**0.004**
HBV (%)	27 (18.2)	17 (16.2)	10 (23.3)	0.351
HCV (%)	12 (8.1)	7 (6.7)	5 (11.6)	0.331
HBV and HCV co-infection (%)	4 (2.7)	2 (1.9)	2 (4.7)	0.580

- HDL- High Density Lipoprotein, LDL- Low Density Lipoprotein - Mean CIMT = mean of the maximum IMT from the far wall of the distal common carotid artery, the carotid bifurcation and the proximal internal carotid on the right and left sides (mean of 6 sites). - Mean CIMT (CCA and BULB) = mean of the maximum IMT from the far wall of both the distal common carotid artery and the carotid bifurcation on the right and left sides (mean of 4 sites).

**Figure 1:**
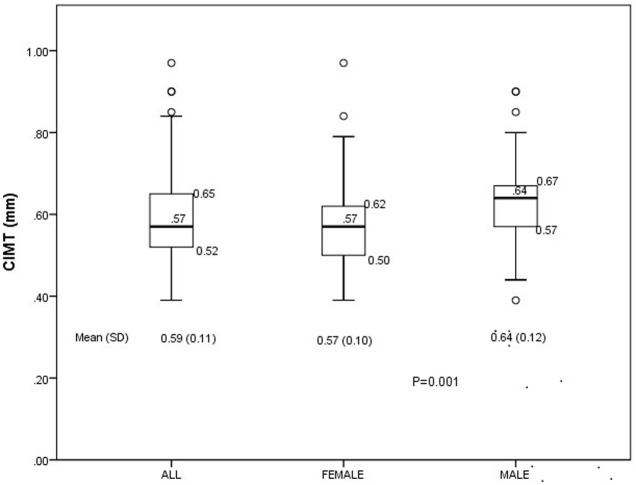
Box-Whisker´s plot of distribution of CIMT

**Figure 2:**
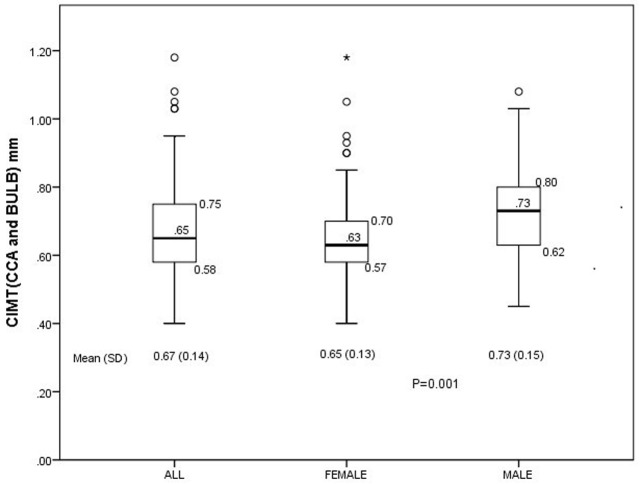
Box-Whisker´s plot of distribution of CIMT (CCA and BULB)

The distribution of the CIMT in the study participants is displayed in Figure 1. The overall Mean (SD) CIMT of all study participants as defined in this study was 0.59 (0.11) mm but this was significantly higher in male [0.64 (0.12) mm] compared to females [0.57 (0.10) mm], p = 0.001. The mean (SD) of CIMT as computed from the CCA and BULB measurements [CIMT (CCA and BULB)] was 0.67 (0.14) mm while the overall prevalence of SCA using this measurement was 18.2%. The distribution of the CIMT (CCA and BULB) in the study participants is shown in Figure 2.

A comparison of socio-demographic and bio-clinical characteristics of participant with SCA and those with normal CIMT in (Table 2) showed that male HIV-infected individuals were significantly more likely to have SCA than females (p = 0.014). The mean (SD) age of individuals with SCA [(52.5 (4.8)] years was higher than those with normal CIMT [41.2 (9.2) years]; p < 0.001. Also, individuals with SCA were more likely to be hypertensive (p < 0.001) with higher systolic BP (p < 0.001) and diastolic BP (p = 0.013) compared to those with normal CIMT. The biochemical parameters (glucose, lipids and hsCRP) and BMI were not significantly different (p = 0.003) in both groups although HIV-infected individuals with SCA were more likely to have metabolic syndrome than those with normal CIMT. Among HIV-infected adults with SCA and normal CIMT, there was no significant difference in HIV-related parameters such as known duration of HIV infection, duration of ARV uses, ART regimen (first line vs second line), Nadir CD4 count, latest CD4 count and hepatitis B and C infections. Also, in this study, SCA was related to history of DM, current smoking status, alcohol use, sedentary lifestyle and family history of stroke (p > 0.05).

**Table 2:** comparison of different variables HIV-infected subjects with subclinical atherosclerosis (SCA) and normal CIMT

Variables	All participants n=148	Normal (CIMT <0.78 mm) n=137 (92.6%)	SCA (CIMT ≥0.78mm) n=11 (7.4%)	P-Value
**Demographic data**				
Gender: Female: male (%)	105 (70.9); 43 (29.1)	101 (73.7); 36 (26.3)	4 (36.4); 7(63.6)	**0.014**
Age (years); mean (SD)	42.1 (9.4)	41.2 (9.2)	52.5 (4.8)	**<0.001**
**Medical history**				
Hypertension (%)	41 (27.7)	32 (23.4)	9(81.8)	**<0.001**
Diabetes mellitus (%)	5 (3.4)	4 (2.9)	1 (9.1)	0.324
Family history of stroke (%)	11 (7.4)	9 (6.6)	2 (18.2)	0.191
Smoking status (%)	7 (4.7)	7 (5.1)	0 (0)	1.000
Alcohol (%)	19 (12.8)	17 (12.4)	2 (18.2)	0.634
Sedentary life>	26 (17.6)	25 (18.2)	1 (9.1)	0.690
**Biophysical parameters**				
Systolic BP (SBP) mmHg (mean; SD)	124.4 (20.7)	122.5 (9.5)	147.0 (20.8)	**<0.001**
Diastolic BP (SBP) mmHg (mean; SD)	78.9 (10.9)	78.4 (10.6)	86.8 (12.6)	**0.013**
BMI (kg/m^2^) (median; IQR)	25.4 (22.2-28.5)	25.4 (22.1-28.4)	26.7 (23.1-29.6)	0.273
**Biochemical parameters**				
Fasting plasma glucose (mmol/L) (mean;SD)	5.3 (1.3)	5.2 1.3)	5.3 (1.3)	0.610
Total cholesterol (mmol/L) (mean;SD)	5.0 (1.3)	5.2 (1.3)	5.5 (0.9)	0.505
Triglyceride (mmol/L) (mean;SD)	1.2 (0.9)	1.2 (0.9)	1.4 (1.0)	0.735
HDL cholesterol (mmol/L) (mean;SD)	1.0 (0.4)	1.1 (0.4)	1.0 (0.3)	0.853
LDL cholesterol (mmol/L) (mean;SD)	2.5 (0.9)	2.5 (0.9)	2.2 (1.1)	0.422
HsCRP (mg/L)(median; IQR)	2.8 (0.8-5.7)	2.9 (1.0-5.9)	3.2 (2.2 -4.2)	0.242
Metabolic syndrome (IDF) (%)	57 (38.5)	48 (35.0)	9 (81.8)	**0.003**
**HIV-Related parameters**				
Known duration of infection (years) (median; IQR)	5.0 (3.0 -5.0)	5 (3-10)	8 (2-11)	0.607
Duration of ARV (years) (median; IQR)	5.0 (3.0 -9.0)	5 (3-9)	3 (2-7)	0.343
Receiving first-line ARV; second-line ARV (%)	138 (93.2); 10 (6.8)	129(94.2) ; 8(5.8)	9(81.8); 2(18.2)	0.163
Nadir CD4 count (cells/ul) (median; IQR)	168 (100.5-244.5)	167(95-258.5)	176(113-209)	0.861
Latest CD4 count (cells/ul) (median; IQR)	448.0 (307.3-607.5)	449 (307.5-609)	401(290-489)	0.624
HBV (%)	27 (18.2)	26 (19.0)	1(9.1)	0.367
HCV (%)	12 (8.1)	10(7.3)	2(18.2)	0.220
HBV and HCV co-infection (%)	4 (2.7)	4(2.9)	0(0)	0.732

Table 3 shows the crude and adjusted odds ratio of factors associated with SCA following binary logistic regression analysis. Elevated blood glucose, lipids and high-sensitivity C-reactive protein, body mass index (BMI), HIV-related parameters (duration of HIV infection, antiretroviral regimen, CD4+ cell count), current smoking status, alcohol use, were not significantly associated with subclinical atherosclerosis (p > 0.05). Male gender [OR(95%CI=4.91 (1.36-17.77)], age [OR(95%CI)=1.14 (1.06-1.23)], hypertension [OR(95%CI=14.4 (3.03-71.86)] and metabolic syndrome [OR(95%CI=8.34 (1.73-40.18)] were significantly associated with SCA at bivariate analysis. After adjusting for age, sex and antiretroviral regimen, only increasing age [Adjusted Odds Ratio (AOR) (95% confidence interval (CI)] = 1.12 (1.01-1.25)] and hypertension [AOR (95%CI) = 10.67 (1.31-87.18)], remained as independent predictors of subclinical atherosclerosis.

**Table 3:** crude and adjusted odds ratios of factors associated with subclinical atherosclerosis using binary logistic regression analysis

Variables	Bivariate Analysis		Multivariate Analysis	
	Crude OR (95%CI)	P-Value	Adjusted OR (95%CI)	P-Value
**Demographic data**				
Gender (Male)	4.91 (1.36-17.77)	**0.009**	1.99 (0.39-10.22)	0.411
Age (years); mean (SD)	1.14 (1.06-1.23)	**0.001**	1.12 (1.01-1.25)	**0.044**
**Medical history**				
Hypertension (%)	14.4 (3.03-71.86)	**<0.001**	10.67 (1.31-87.18)	**0.027**
Diabetes mellitus (%)	3.33 (0.34-32.63)	0.276		
Family history of stroke (%)	3.16 (0.59-16.87)	0.158		
Smoking status (%)				
Alcohol (%)				
Sedentary life	0.45 (0.06-3.67)	0.443		
**Bioclinical parameters**				
BMI (kg/m^2^) (mean; SD)	1.07 (1.00-1.45)	0.087		
Metabolic syndrome (IDF) (%)		**0.002**	3.23 (0.40-25.84)	0.270
Fasting plasma glucose (mmol/L) (mean;SD)	1.14 (0.77-1.68)	0.507		
Total cholesterol (mmol/L) (mean;SD)	0.92(0.56-1.50)	0.732		
Triglyceride (mmol/L) (mean;SD)	1.19 (0.67-2.14)	0.553		
HDL cholesterol (mmol/L) (mean;SD)	0.85 (0.15-4.86)	0.851		
LDL cholesterol (mmol/L) (mean;SD)	0.75 (0.38-1.50)	0.419		
HsCRP (mg/L) (median; IQR)	1.00 (0.94-1.05)	0.507		
**HIV-Related parameters**				
Known duration of infection (years)	1.07 (0.92-1.23)	0.389		
Duration of ARV (years)	0.92 (0.78-1.10)	0.352		
Receiving first-line ARV	0.28 (0.05-1.51)	0.117		
Nadir CD4 count	1.00 (0.99-1.00)	0.624		
Latest CD4 count (cells/ul)	1.00 (0.99-1.00)	0.702		

Multivariate analysis involved variables with P<0.02 in the Bivariate Analysis

## Discussion

The prevalence of SCA among HIV-infected adults in this study was found to be 7.4%. In the context of a setting with high HIV burden such as in Nigeria, this represents a significant population with increased CVD risk. In similar largely female dominated HIV- infected population in South Africa and Uganda, Schoffelen *et al*. and Ssinabulya *et al*. reported prevalence of SCA at 12% and 18% respectively [11,13]. The median age in both studies were comparable with ours. However, whereas in this study CIMT was computed from the mean IMT of the CCA, BULB AND ICA segments, CIMT was computed from the mean IMT of CCA and the BULB segment in the south African study, and from the CCA segment in the Ugandan study [11,13]. These differences in prevalence rates underscore the lack of uniformity in CIMT measurement protocols which have been highlighted in several literatures making comparison difficult. It is noteworthy that using a similar scan and imaging analysis protocol as that used in the South African study, we observed a higher median (IQR) CIMT [0.65 (0.58-0.75] mm compared to [0.59 (0.52-0.68)] mm obtained in their study and this was also maintained across the male and female divide. Also, the prevalence of SCA of 18.2% obtained was higher than the rate in South Africa but similar to the Ugandan prevalence. In comparison to the findings in other continents, median (IQR) CIMT was slightly higher [0.57 (0.52-0.65)] than as observed in a Brazilian cohort of HIV-infected individuals [0.54 (0.49-0.62)] [29]. The prevalence rate obtained in this study was lower than reported among HIV-infected populations in European and Asian studies even at more stringent CIMT of between 0.80-0.90 mm. Prevalence of SCA of 41.7%, 56.4% and 41.3% have been reported in different HIV-infected cohorts in Italy, Spain and Malaysia respectively [30-32]. These differences in prevalence rates compared to those obtained in SSA may denote variations in the profile of modifiable and non- modifiable risk factors for CVDs. Also, CIMT have been shown to be higher in males compared to females and this was consistent in this study. Higher male proportion (60-70%) reported in the Italian, Spanish and Malaysian studies cited above may contribute to the higher prevalence of SCA reported in these studies [30-32]. CIMT is also positively correlated with age. This study had a similar age profile with the other referenced as demonstrated by comparable median age of the study participants. Remarkably, none of the 40% of the participants <40 years had SCA. The result from this study showed that for each unit (1 year) increase in age, the odds of having SCA increased by 14%. The association between age and SCA remained significant even after adjusting for sex. This finding is consistent with previous reports on the association of age and SCA or CIMT [11,13,25,33,34].

The association between traditional and non-traditional risk factors for CVD and SCA in HIV-infected individuals was examined in this study. Hypertension stood out as the main predictor of SCA. The mean SBP and DBP were significantly higher in HIV-infected individuals compared to those with normal CIMT. Participants with hypertension were 14 times more likely to have SCA compared to those with normal blood pressure. Hypertension also remained an independent predictor of SCA in the multivariate analysis. Hypertension was demonstrated as risk factor for SCA in almost all the studies reviewed. Our result showed that there was no significant association between lipid profile (total cholesterol, triglyceride, HDLc and LDLc), diabetes mellitus, BMI, current smoking status, current alcohol use, sedentary lifestyle and family history of stroke with SCA. These findings show some contrast with the report from the early studies conducted in Africa. In Uganda, in addition to Hypertension and advancing age, only increased BMI (obesity), total cholesterol, LDLc and active smoking were significantly associated with SCA [11]. In South Africa, traditional cardiovascular risk factors were associated with increased CIMT. Age, male gender, previous CVD event, hypertension, BMI, obesity, diabetes mellitus, total cholesterol, LDLc and metabolic syndrome were significantly and independently associated with increased CIMT [12]. Several researchers on HIV-infected individuals in western countries have also demonstrated association of traditional risk factors such as dyslipidaemia, obesity, metabolic syndrome, advancing age and atherosclerosis indicated by increased CIMT [34-39]. Like elsewhere in SSA [11,13], smoking was not linked to SCA as seen in western studies [34,40]. The relationship between hsCRP, a marker of inflammation and SCA was also not significant. This was consistent with previous studies on HIV-infected individuals both in Africa and in other continents that have shown poor correlation between marker of inflammation such as hsCRP and CIMT [11,13,34]. Forty-eight percent of our subjects had hsCRP of ≥3.0 mg/L while 54% of the study participants in the Ugandan study had hsCRP of ≥3.0 mg/L. The relatively high hsCRP levels may be in keeping with persistent inflammation due to higher tendency of exposure to numerous infectious agents, a likely scenario in the context of HIV infection and the African environment. This calls to question the use of similar cut-off values for predicting CVDs as in western countries. This confounding situation may have obscured our understanding of the use of hsCRP as a predictor of CVDs in the setting of HIV in Africa.

There are conflicting reports with respect to the role of HIV-related factors in relation to SCA and increased CIMT. Several studies have reported an association between HIV-related parameters and progression of CIMT. Time of diagnosis, elevated CD4 count, increased viral load, duration of non-nucleoside reverse transcriptase inhibitors, duration of protease inhibitors use are among HIV-related factors that have been reported to affect CIMT [32,34,41-45]. However, in the current study as in the previous African studies [11,13], none of these factors were significantly associated with SCA. This study to our knowledge is one of the first to examine cardiovascular risk factors in a large HIV cohort in the context of a cardiovascular endpoint such as subclinical atherosclerosis in Nigeria and Western African sub-region. This complements reports from Southern and Eastern Africa [11,13]. It is evident that whereas the prevalence of SCA is comparable across Africa, the risk factors vary considerably. Unlike in South and East Africa, traditional CVD risk factors like dyslipidaemia and obesity appear not to be significant in the progression of atherosclerotic changes in Nigerian population. This study also examined a wide spectrum of risk factors including the roles of inflammatory makers such as hsCRP and HIV-related factors in CVD progression. However, this study had some limitations. In the absence of universally acceptable definition for SCA, a surrogate cut-off of ≥0.78 mm for CIMT was adopted to define SCA in this study. Although this cut-off has been widely used even in previous studies including in Africa, it would have been better to use a cut-off derived from a healthy Nigerian population. Nevertheless, we believe the chosen cut-off is stringent-enough considering that a widely referenced study has estimated that a healthy person reaches this value at age 76 years [28]. Owing to low frequency of some risk factors such as smoking and diabetes mellitus in this study population, this study may have lacked sufficient power to properly elucidate the relationship between these risk factors and SCA. Also, the descriptive cross-sectional nature of the study does not establish causality. Although demonstration of association in this study is a critical step in understanding the relationship between CVD risk factors and CVD progression in HIV patients, we advocate future longitudinal prospective studies to better appreciate the magnitude of the impact of these risk factors in CVD progression in HIV-infected population.

## Conclusion

This study has shown that SCA is common in Nigerian HIV-infected adults. Advancing age and hypertension were found to be significantly associated with SCA. Traditional CVD risk factors like abnormal lipid fractions, diabetes mellitus and obesity individually were not associated with CIMT in this population. We recommend early identification and management of hypertension as part of standard of care for HIV-infected adults in Nigeria.

### What is known about this topic

- Cardiovascular risk factors abound in HIV-infected individuals;
- Traditional CVD risk factors and HIV-related factors have been shown to be associated with subclinical atherosclerosis in HIV-infected individuals in many western populations.

### What this study adds

- The study has provided us with the burden of subclinical atherosclerosis in Nigerian HIV-infected individuals which was hitherto undocumented;
- Data obtained indicates that HIV-related factors and some traditional risk factors of CVD (like abnormal lipid fractions, diabetes mellitus and obesity) were not associated with atherosclerotic changes in HIV-infected individuals;
- Hypertension and advancing age are the independent predictors of subclinical atherosclerosis in Nigerian HIV-infected individuals.
